# Supplementary material for: Identification of Freezing-Responsive microRNAs and Their Targets in Chinese Jujube by Small RNA and Degradome Sequencing
Source: Int J Mol Sci. 2026 Sep 4;27(17):7912. doi: 10.3390/ijms27177912 (PMC13566890; doi:10.3390/ijms27177912)
Supplement: Supplementary file 1 [file ijms-27-07912-s001.zip › Supplementary Material S2/novel_miR_79.pdf]

Provisional ID	:	novel_miR_79
Score total	:	0.4
Score for star read(s)	:	-1.3
Score for read counts	:	0
Score for mfe	:	0.1
Score for randfold	:	1.6
Score for cons. seed	:	
Total read count	:	9228
Mature read count	:	9159
Loop read count	:	33
Star read count	:	36

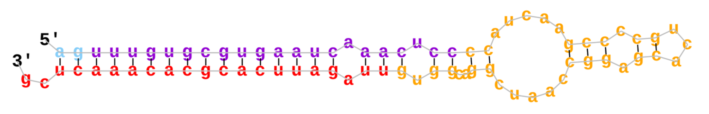

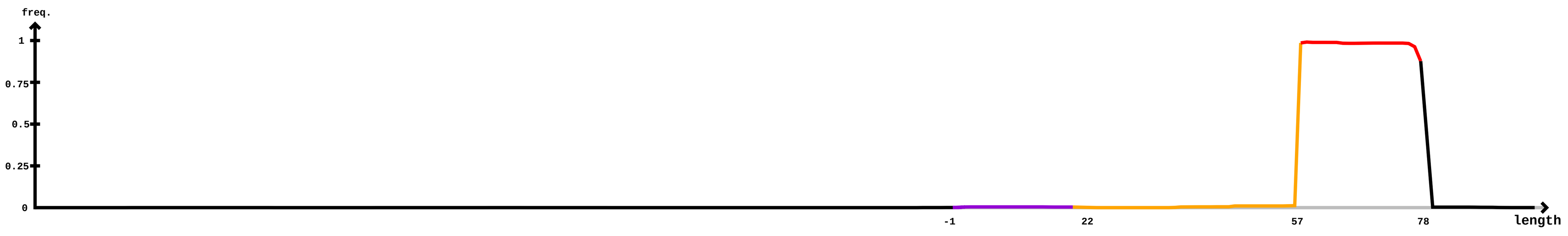

Star Mature

```
5'- uuucaaaaccauuucagugucuauugccccuauuuuauuuuuuuauuuuuuuuuauuuauuuucucgucuaccuuuuuuuuuuucuuuuuauauauauuaacucucuccauuuuucucucuuuuucgauuagaagaugggacacaucucaaguuugugcgugaaucaaacuccccaucaagccccgucacgaggccaaucggacggugguuagauucacgcacaaacucgcgaucugucuccucuaaaaa -3' obs
    uuucaaaaccauuucagugucuauugccccuauuuuauuuuuuuauuuuuuuuuauuuauuuucucgucuaccuuuuuuuuuuucuuuuuauauauauuaacucucuccauuuuucucucuuuuucgauuagaagaugggacacaucucaaguuugugcgugaaucaaacuccccaucaagccccgucacgaggccaaucggacggugguuagauucacgcacaaacucgcgaucugucuccucuaaaaa     exp
    .............((((....))))..................................................................((((((((...((......))...))))))))((((.(((.(.((((((((((((((((.(((.((((.....(((.((...)).))).....))..)).))).))))))))))))))))).).))).)))).........
```

read	reads	mm	sample
cagugucuauugccccuauuuua	2	0	JCK-2
auuuauuuucucgucuaccuuuuuu	1	0	LT-1
Cuuuuuuuuuucuuuuuaua	1	1	LT-2
ucucaaguuugugcgugaauc	5	0	LT-2
aaguuugugcgugaauca	2	0	LT-1
uuugugcgugaaucaaacucc	9	0	LT-2
uuugugcgugaaucaaacucc	2	0	JCK-3
uuugugcgugaaucaaacucc	5	0	LT-1
uuugugcgugaaucaaacucc	5	0	LCK-3
uuugugcgugCaucaaacucc	1	1	LT-2
uuugugcgugaaucaaacucc	1	0	JCK-1
uuugugcgugaaucaaacucc	7	0	LT-3
uugugcgugaaucaaacucc	2	0	JCK-2
uugugcgugaaucaaacucc	1	0	JCK-3
uugugcgugaaucaaacucccc	1	0	LT-3
cacgaggccaaucggacggugu u	2	0	LT-2
cacgaggccaaucggacggugu u	4	0	LCK-3
cacgaggccaaucggacgguguuagauuc	1	0	JCK-1
cacgaggccaaucggacgguguuagauuc	3	0	JCK-3
cacgaggccaaucggacgguguuagauuc	1	0	JCK-2
acgaggccaaucggacggu	3	0	LT-3
acgaggccaaucggacggugu	1	0	LT-3
acgaggccaaucggacgguguu	10	0	LCK-2
acgaggccaaucggacgguguu	12	0	LCK-1
acgaggccaaucggacgguguuaga	1	0	LT-1
cgaggccaaucggacgguguuagauucacg	2	0	LT-2
gaggccaaucggacgguguuagauucacg	1	0	LT-2
gaggccaaucggacgguguuagauucacgc	1	0	LT-1
aggccaaucggacgguguuagauuc	1	0	LT-1
aggccaaucggacgguguuagauucacg	1	0	LT-1
ccaaucggacgguguuagauucacgcU	2	1	JCK-3
ccaaucggacgguguuagauucacgcaA	1	1	JCK-1
ccaaucggacgguguuagauucacgcaca	1	0	JT-3

Star Mature

uuucaaaaccauuucagugucuauugcccccuauuuuauuuuuuuauuuuuuuuuauuuauuuucucgucuaccuuuuuuuuuuucuuuuuauauauauuaacucucuccauuuuucucucuuuuucgauuagaagaugggacacaucucaaguuugugcgugaaucaaacucccaucaagccccgucacgaggccaaucggacgguguuagauucacgcacaaacucgcgaucugucuccucuaaaaa

aucggNcgguguuagauu	1	1	LCK-1
aucggacgguguuagauu	23	0	LCK-1
aucggaAgguguuagauu	1	1	LCK-2
aucggacgguguuagauu	20	0	LCK-2
aucggacgguguuagauucacgcaca	1	0	LT-3
uguuagauucacgcacaaacuc	1	0	LT-3
uguuagauucacgcacaaacuc	2	0	LT-1
uguuagauucacgcacaaacucg	1	0	JCK-3
uguuagauucacgcacaaacucg	1	0	LT-1
uguuagauucacgcacaaacucg	2	0	JCK-2
uguuagauucacgcacaaacucgc	1	0	LT-3
guuagauucacgcacaaacucA	2	1	JCK-1
Cuuagauucacgcacaaacucg	2	1	JCK-2
Cuuagauucacgcacaaacucg	3	1	LT-1
guuagauucacgcacaaacucA	1	1	JCK-3
Cuuagauucacgcacaaacucg	2	1	JCK-3
Cuuagauucacgcacaaacucg	1	1	LT-3
uuagauucacgcacaaac	9	0	LCK-3
uuagauucacgcacaaac	5	0	LT-1
uuagauucacgcacaaac	5	0	LT-3
uuagauucacgcacaaac	2	0	JCK-2
uuagauucacgcacaaac	1	0	JCK-1
uuagauucacgcacaaacu	23	0	LCK-2
uuNgauucacgcacaaacu	1	1	LT-2
uuagauucacgcacaaacu	24	0	LT-3
uuagauucacgcacaaacu	31	0	LCK-3
uuagauucacgcacaaacu	33	0	LT-1
uuagauucacgcacaaacu	14	0	JCK-3
uuagauucacgcacaaaUu	5	1	LCK-3
uuagauucacgcacaaacu	22	0	LT-2
uuagauucacgcacaaacu	21	0	LCK-1
uuagauucacgcacaaacu	4	0	JCK-1
uuagauucacgcacaaacu	5	0	JCK-2
uuagauucacgcacaaacuc	19	0	JCK-3
uuCgauucacgcacaaacuc	1	1	LCK-3
uuagauucacgcacaaacuc	57	0	JT-1
uuagauucacgcacaaacuc	1	0	JT-2
uuagauucacgcCcaaacuc	1	1	LT-3
uuagauucCcgcacaaacuc	1	1	LT-1
uuagauucacgcacaaacuc	37	0	LCK-2
uuagauucacgcacaaacuc	27	0	JCK-1
uuagauucacgcacaaacuc	156	0	LT-3
uuagCuucacgcacaaacuc	1	1	LT-3
uuagauucacgcacaaacuU	2	1	LT-1
uuagauucacgcacaaacuU	2	1	LT-2
uuagauucacgcacaaacuc	31	0	JCK-2
uuCgauucacgcacaaacuc	1	1	LT-1
uuagauucacgcacaaacuc	142	0	LCK-3
uuagauucacgcacaaacuc	162	0	LT-2
uuagauucacgcacaaacuc	4	0	JT-3
uuagauucacgcacaaacuU	7	1	LCK-3
uuagCuucacgcacaaacuc	1	1	LT-2
uuagauucacgcacaaacuU	5	1	LT-3
uuagCuucacgcacaaacuc	1	1	LT-1
uuagauucacgcacaaacuc	33	0	LCK-1
uuagauucacgcacaaacuc	104	0	LT-1
uuagauucacgcacaaacucg	1306	0	JCK-2
uuagauucacgcacaGacucg	2	1	LT-1
uuagauucCcgcacaaacucg	3	1	JCK-2
uuagauucCcgcacaaacucg	4	1	LT-3
uuagauucacgcacaaCcucg	2	1	LT-3
uuCgauucacgcacaaacucg	1	1	LCK-3
uuagauucacgcacaaacucA	1	1	JCK-3
uuagauucacgcacaaCcucg	1	1	LT-2
uuCgauucacgcacaaacucg	1	1	LCK-2
uuagauucacgcacaaacucA	1	1	LCK-3
uuagauucacgcaAaaacucg	1	1	JCK-2
uuagauucacgcacaaacucU	14	1	LT-3
uuNgauucacgcacaaacucg	3	1	LT-2
uuagauucacgcacaaacucg	1410	0	LT-3

Star Mature

```
uuucaaaaccauuucagugucuauugcccccuauuuuauuuuuuuauuuuuuuuuauuuauuuucucgucuaccuuuuuuuuuuucuuuuuauauauauuaacucucuccauuuuucucucuuuuucgauuagaagaugggacacaucucaaguuugugcgugaaucaaacuccccaucaagccccgucacgaggccaaucggacgguguuagauucacgcacaaacucgcgaucugucuccucuaaaaa
.................................................................................................................................................................................................................uuagauucacgcacaaacucU....................  7     1  LCK-3
.................................................................................................................................................................................................................uuagauucacgcCcaaacucg....................  1     1  LT-3
.................................................................................................................................................................................................................uuagauucacgcacaaacucU....................  26    1  LT-2
.................................................................................................................................................................................................................uuagauucacgcacaaacuAg....................  2     1  LT-2
.................................................................................................................................................................................................................uuagauucacgcacaaacucg....................  628   0  LCK-3
.................................................................................................................................................................................................................uuagauucacgcacaaCcucg....................  1     1  JCK-3
.................................................................................................................................................................................................................uuagaNucacgcacaaacucg....................  1     1  JCK-3
.................................................................................................................................................................................................................uuagauucacgUacaaacucg....................  1     1  LT-1
.................................................................................................................................................................................................................uuagauucacAcacaaacucg....................  1     1  LT-2
.................................................................................................................................................................................................................uuagauucacgcacaaaGucg....................  1     1  JCK-1
.................................................................................................................................................................................................................uuagauucacgcacaCacucg....................  1     1  JCK-3
.................................................................................................................................................................................................................uuagCuucacgcacaaacucg....................  1     1  LCK-3
.................................................................................................................................................................................................................uuagauucacgcacaaacucg....................  730   0  JCK-1
.................................................................................................................................................................................................................uuagauucacgcGcaaacucg....................  1     1  JCK-3
.................................................................................................................................................................................................................uuagCuucacgcacaaacucg....................  2     1  JT-1
.................................................................................................................................................................................................................uuagauucacgcacaaacucg....................  1198  0  LT-1
.................................................................................................................................................................................................................uuCgauucacgcacaaacucg....................  2     1  JCK-2
.................................................................................................................................................................................................................uuagauucacgcacaaacucU....................  8     1  JCK-1
.................................................................................................................................................................................................................uuagauucacgcacaaacucg....................  91    0  JT-1
.................................................................................................................................................................................................................uuagauucacgcacaaacucN....................  1     1  JCK-1
.................................................................................................................................................................................................................uuagauuUacgcacaaacucg....................  2     1  LT-3
.................................................................................................................................................................................................................uuagauucacgcacaaacNcg....................  1     1  JCK-1
.................................................................................................................................................................................................................uuagCuucacgcacaaacucg....................  7     1  LT-2
.................................................................................................................................................................................................................uuagauucacgcacaaacucg....................  988   0  JCK-3
.................................................................................................................................................................................................................uuCgauucacgcacaaacucg....................  1     1  LT-1
.................................................................................................................................................................................................................uuagauucacAcacaaacucg....................  1     1  JCK-1
.................................................................................................................................................................................................................uuagauucacgcacaCacucg....................  1     1  LT-3
.................................................................................................................................................................................................................uCagauucacgcacaaacucg....................  1     1  JCK-2
.................................................................................................................................................................................................................uuagauucacgcUcaaacucg....................  1     1  JCK-1
.................................................................................................................................................................................................................uuagauucacgcacaaacucg....................  1110  0  LT-2
.................................................................................................................................................................................................................uuagauucacgcGcaaacucg....................  1     1  LT-1
.................................................................................................................................................................................................................uuagauucacgcacaCacucg....................  2     1  LT-2
.................................................................................................................................................................................................................uuagauucCcgcacaaacucg....................  2     1  LCK-3
.................................................................................................................................................................................................................uuagauucacgcacaaacucU....................  11    1  LT-1
.................................................................................................................................................................................................................uNagauucacgcacaaacucg....................  2     1  JCK-2
.................................................................................................................................................................................................................uuagauucacgcUcaaacucg....................  1     1  JCK-3
.................................................................................................................................................................................................................uuagauucacgcacaaacucg....................  22    0  JT-3
.................................................................................................................................................................................................................uuagauucacgcacaaacucg....................  168   0  LCK-1
.................................................................................................................................................................................................................uuagauucacgcacaaCcucg....................  2     1  LT-1
.................................................................................................................................................................................................................uuagauucacgcacaaacucA....................  3     1  LT-1
.................................................................................................................................................................................................................uuagauucacgcacaCacucg....................  1     1  LT-1
.................................................................................................................................................................................................................uuCgauucacgcacaaacucg....................  11    1  LT-2
.................................................................................................................................................................................................................uuagauucacgcacaaacucg....................  181   0  LCK-2
.................................................................................................................................................................................................................uuagauucacgAacaaacucg....................  1     1  LT-1
.................................................................................................................................................................................................................uuagCuucacgcacaaacucg....................  5     1  LT-3
.................................................................................................................................................................................................................uuCgauucacgcacaaacucg....................  4     1  LT-3
.................................................................................................................................................................................................................uuCgauucacgcacaaacucg....................  2     1  JCK-1
.................................................................................................................................................................................................................uuagauucCcgcacaaacucg....................  4     1  LT-2
.................................................................................................................................................................................................................uuagauucCcgcacaaacucg....................  1     1  JCK-3
.................................................................................................................................................................................................................uuagauucCcgcacaaacucg....................  1     1  LT-1
.................................................................................................................................................................................................................uuagauucacgcaNaaacucg....................  1     1  LCK-1
.................................................................................................................................................................................................................uuagauucacgcaUaaacucg....................  1     1  JCK-1
.................................................................................................................................................................................................................uuagCuucacgcacaaacucg....................  2     1  LT-1
.................................................................................................................................................................................................................uuagauucacgcacaaacucg....................  18    0  JT-2
.................................................................................................................................................................................................................uuagauucacgcaUaaacucg....................  1     1  LT-1
.................................................................................................................................................................................................................uuagauucacgcacaaacucU....................  6     1  JCK-2
.................................................................................................................................................................................................................uuagauucGcgcacaaacucg....................  1     1  JCK-3
.................................................................................................................................................................................................................uuagauucacgcacaaaAucg....................  1     1  LT-1
.................................................................................................................................................................................................................uuagauucacgNacaaacucg....................  1     1  JT-1
.................................................................................................................................................................................................................uuagCuucacgcacaaacucg....................  2     1  JCK-3
.................................................................................................................................................................................................................uuagauucacgcacaCacucg....................  1     1  JCK-2
.................................................................................................................................................................................................................uuagauucacgcacaaacucU....................  8     1  JCK-3
.................................................................................................................................................................................................................uuagauucacgcacGaacucg....................  1     1  JCK-2
.................................................................................................................................................................................................................uuagauucacgcacaaacucgA...................  9     1  LT-2
.................................................................................................................................................................................................................uuagauucacgcacaaacucgA...................  16    1  LT-1
.................................................................................................................................................................................................................uuagauucacgcacaaacucgA...................  1     1  JCK-2
.................................................................................................................................................................................................................uuagauucacgcacaaacucgA...................  1     1  JCK-1
.................................................................................................................................................................................................................uuagauucacgcacaaacucgA...................  6     1  LT-3
.................................................................................................................................................................................................................uuagauucacgcacaaacucgA...................  11    1  LCK-3
.................................................................................................................................................................................................................uuagauucacgcacaaacucgG...................  1     1  LT-1
```

Star Mature

```
uuucaaaaccauuucagugucuauugcccccuauuuuauuuuuuuauuuuuuuuuauuuauuuucucgucuaccuuuuuuuuuuucuuuuuauauauauuaacucucuccauuuuucucucuuuuucgauuagaagaugggacacaucucaaguuugugcgugaaucaaacuccccaucaagccccgucacgaggccaaucggacggugCuuagauucacgcacaaacucgcgaucugucuccucuaaaaa
.................................................................................................................................................................................................................uagauucacgcacaaacu......................   1   0   LT-2
.................................................................................................................................................................................................................uagauucacgcacaaacuc.....................   2   0   LT-3
.................................................................................................................................................................................................................uagauucacgcacaaacuc.....................   1   0   LCK-3
.................................................................................................................................................................................................................uagauucacgcacaaacucg....................   2   0   JCK-3
.................................................................................................................................................................................................................uagauucacgcacaaacucg....................   8   0   LT-3
.................................................................................................................................................................................................................uagauucacgcacaaacucg....................   4   0   LT-1
.................................................................................................................................................................................................................uagauucacgcacaaacucg....................   2   0   JCK-1
.................................................................................................................................................................................................................uagCuucacgcacaaacucg....................   1   1   LT-3
.................................................................................................................................................................................................................uagauucacgcacaaacucg....................   7   0   JCK-2
.................................................................................................................................................................................................................uagauucacgcacaaacucgc...................   7   0   JCK-2
.................................................................................................................................................................................................................uagauucacgcacaaacucgc...................   2   0   JCK-3
.................................................................................................................................................................................................................uagauucacgcacaaacucgc...................   2   0   LT-1
.................................................................................................................................................................................................................uagauucacgcacaaacucgc...................   5   0   LT-3
.................................................................................................................................................................................................................uagauucacgcacaaacucgc...................   5   0   JCK-1
..................................................................................................................................................................................................................agauucacgcacaaacuc.....................   2   0   JCK-1
..................................................................................................................................................................................................................agauucacgcacaaacuc.....................   2   0   JCK-3
..................................................................................................................................................................................................................agauucacgcacaaacucg....................   1   0   LT-1
..................................................................................................................................................................................................................agauucacgcacaaacucg....................   2   0   LT-2
..................................................................................................................................................................................................................agauucacgcacaaacucgc...................   1   0   LT-3
..................................................................................................................................................................................................................agauucacgcacaaacucgcg..................   1   0   LT-1
..........................................................................................................................................................................................................................gcacaaacucgcgaucugucucc........   2   0   JCK-1
..........................................................................................................................................................................................................................gcacaaacucgcgaucugucucc........   2   0   JCK-2
..........................................................................................................................................................................................................................gcacaaacucgcgaucugucucc........   2   0   JCK-3
...........................................................................................................................................................................................................................cacaaacucgcgaucugu............   3   0   LCK-3
...........................................................................................................................................................................................................................cacaaCcucgcgaucuguc...........   1   1   LT-2
...........................................................................................................................................................................................................................cacaaacucgcgaucuguc...........   3   0   LT-2
...........................................................................................................................................................................................................................cacaaacucgcgaucugucuc.........   1   0   LT-3
............................................................................................................................................................................................................................acaaacucgcgaucugucu..........   1   0   LT-3
............................................................................................................................................................................................................................acaaacucgcgaucugucucc........   1   0   JCK-1
............................................................................................................................................................................................................................acaaacucgcgaucugucucc........   1   0   LT-1
............................................................................................................................................................................................................................acaaacucgcgaucugucucc........   1   0   LT-3
.............................................................................................................................................................................................................................caaacucgcgaucugucuccu.......   1   0   LT-3
.............................................................................................................................................................................................................................caaacucgcgaucugucuccuc......   1   0   JCK-2
.............................................................................................................................................................................................................................caaacucgcgaucugucuccuc......   1   0   JCK-3
...............................................................................................................................................................................................................................aacucgcgaucugucucc........   1   0   JCK-2
...............................................................................................................................................................................................................................aacucgcgaucugucuccu.......   1   0   LT-3
..................................................................................................................................................................................................................................ucgcgaucugucuccucuaaa..   1   0   LT-3
..................................................................................................................................................................................................................................ucgcgaucugucuccucuaaaa.   1   0   LT-3
.....................................................................................................................................................................................................................................cgaucugucuccucuaaaa.   1   0   LT-1
......................................................................................................................................................................................................................................gaucugucuccucuaaaU.   1   1   LT-3
```
